# Comparison of the fluorescence microscopy Cyscope® with light microscopy for malaria diagnosis in a small and active surveillance in Cameroon

**DOI:** 10.1186/s41182-020-00234-7

**Published:** 2020-07-28

**Authors:** Christian Mbohou Nchetnkou, Hervé Nyabeyeu Nyabeyeu, Loick P. Kojom Foko, Leopold G. Lehman

**Affiliations:** 1 Department of Animal Organisms, Faculty of Science, The University of Douala, P.O. Box 24157, Douala, Cameroon; 2 Department of Biological Sciences, Faculty of Medicine and Pharmaceutical Sciences, The University of Douala, P.O. Box 24157, Douala, Cameroon

**Keywords:** Malaria, Fluorescence microscopy, Diagnosis performances, Company, Cameroon

## Abstract

**Background:**

Malaria has a negative impact on the activities of companies in endemic countries especially in Cameroon. In this regard, an increasingly growing number of companies have started to include management of malarious patients in their health policies. In the present study, we will evaluate the diagnostic performances of a fluorescence microscopy (FM), Cyscope® microscope, in the detection of malaria parasites.

**Methods:**

A cross-sectional study was conducted among employees of two companies of the town of Douala on 21 and 22 March 2017. Sociodemographic information of employees was collected using a questionnaire form. Blood samples of ~ 10 μL were collected by venipuncture for the diagnosis of malaria using FM and light microscopy (LM). Performances of FM with respect to sensitivity (Se), specificity (Sp), positive and negative predictive values (PPV and NPV), positive and negative likelihood rates (PLR and NLR), accuracy, reliability, and Kappa index were calculated using LM as gold standard.

**Results:**

In total, 442 employees, aged 37.8 ± 9.7 years old on average, were included in the study. Prevalence of malaria using FM and LM was 39.2% and 17%, respectively (*p* < 0.01). *Plasmodium falciparum* and *P. vivax* were the two species involved in malaria infection cases. In terms of developmental stages, 68%, 45.3%, and 1.3% of employees carried gametocytes, trophozoites, and schizonts, respectively. Findings on diagnostic performances of FM were as follows: Se = 84%, Sp = 69.95%, PPV = 63.58%, NPV = 95.5%, accuracy = 89.36%, and reliability = 53.95%. Sensitivity of Cyscope® microscope increased as a function of parasitemia with values ranging from 76.92% at parasitemia between 1 and 500 parasites/μL to 91.11% at parasitemia between 501 and 5000 parasites/μL. The geometric mean parasite density was1850 parasites per μL of blood (range 1600–40,000), and most of employees (60.8%) had moderate parasitemia. The performances of FM were similar between febrile and afebrile patients.

**Conclusions:**

This study showed good performances of Cyscope® microscope and outlines that this diagnostic tool could be used in management of malaria at workplace.

## Background

Malaria is a febrile illness infecting humans and primarily caused by parasites transmitted to humans by female mosquitoes belonging to genus *Anopheles* [1]. This parasite belongs to the genus *Plasmodium*, of which five species (*P*. *vivax*, *P*. *malariae*, *P*. *ovale*, *P*. *knowlesi*, and *P*. *falciparum*) are responsible for human malaria. Malaria represents of one the most life-threatening infectious diseases in humans. According to the World Health Organization (WHO), 95% of malaria cases are caused by *P. falciparum*; 228 million cases of malaria are recorded with nearly 435,000 deaths linked to this disease worldwide in 2018 [2]. The sub-Saharan Africa (SSA) region is the most affected by malaria with 93% and 94% of all disease cases and deaths occurred worldwide [2].

The endemicity of this disease in SSA has a negative impact on the professional world [3, 4] as it slows down business productivity [4] and consequently economic growth in several countries [5]. In SSA countries, many control strategies actions, such as the use of long-lasting insecticide-treated mosquito net campaigns [6], indoor residual spraying and sanitation campaigns, have been taken by many countries government and non-governmental organizations to fight against malaria [7–10]. Unfortunately, these measures were taken mainly in the community [6] as well as a small part in the professional environment. However, the diagnostic component has not yet been clearly addressed by governments. To assist government intervention, private companies must play a big role in the fight against malaria through funding and also execute programs for the implementation of sustainable strategies.

In order to reduce or eradicate malaria in the workplace, several strategies have been put in place, namely, sensitization and distribution of insecticide-treated nets (ITNs) [11], the creation of health centers/infirmary to promptly diagnose infection cases [12, 13], and care management. Reliable diagnosis will help to build improved targeted prevention programs [12]. Several diagnostic methods have been developed such as quantitative buffy coat, immunochromatographic rapid diagnostic tests (RDTs), and serological tests [14]. Some companies have adopted the use of conventional microscopy or RDTs in testing for malaria in suspected patients. Unfortunately, these techniques have some drawbacks. For example, light microscopy (LM) is time-consuming and requires the use of quality reagents and skilled microscopist. On the other hand, the immunochromatographic RDTs (1) cannot make a distinction between acute and past infection and (2) can elicit false negative due to bad handling, prozone effect, and recently to deletions in the histidine-rich protein II gene especially for RDTs targeting PfHRP2 protein of *P. falciparum* [15].

**Fig. 1:**
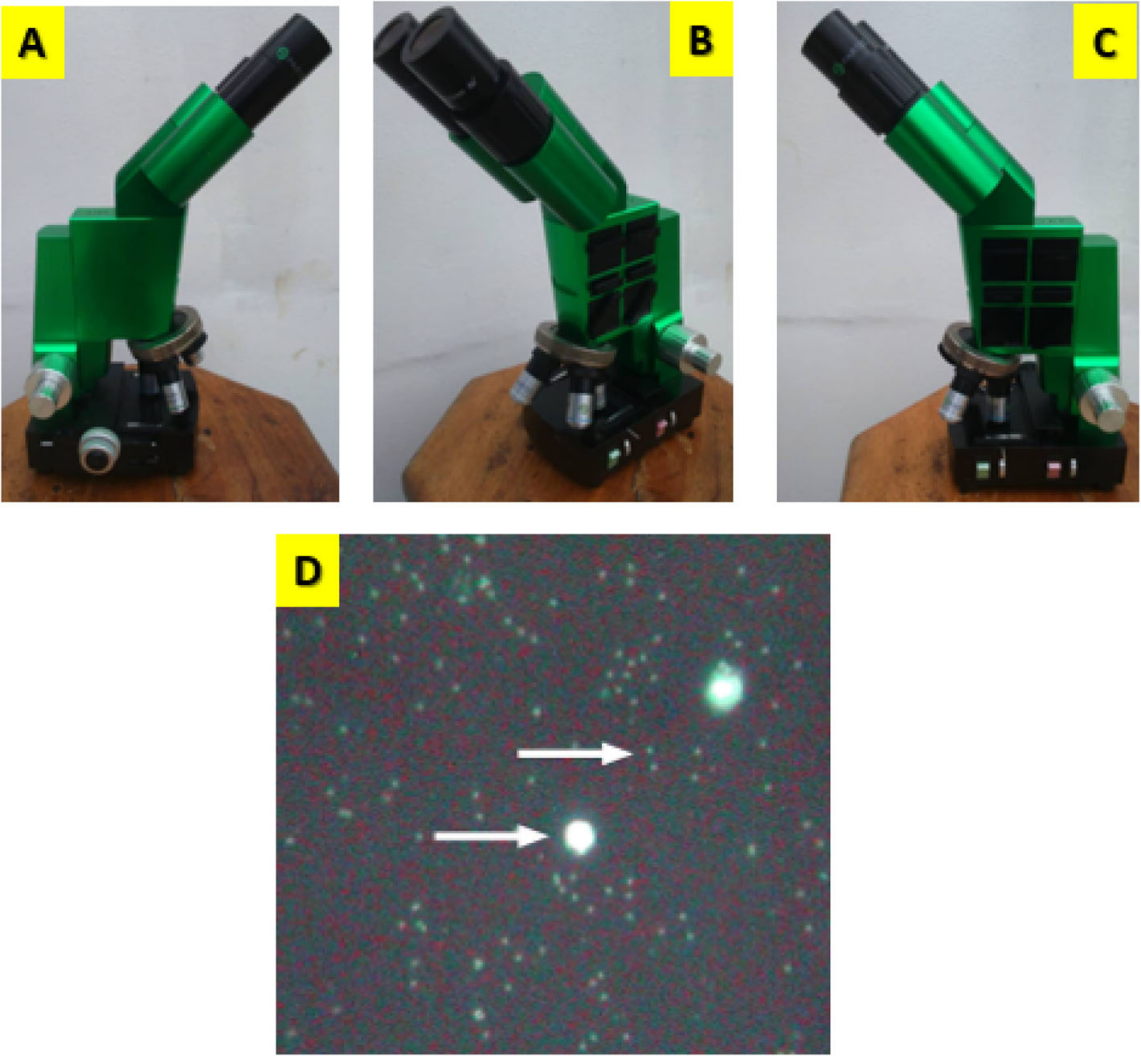
**a**–**c** Cyscope® microscope (Partec-Sysmex, Japan). **d** Positive malaria slides under observation with CyScope®. Bigger spots correspond to WBC and the smaller spots to malaria parasites (Photographs are provided by the authors)

In view of this, there is need for new diagnostic methods that can overcome the abovementioned limitations of LM and immunochromatographic RDTs. Fluorescence-based methods may be used as an alternative especially that based on the use of 4′,6-diamidino-2-phenylindole (DAPI) as fluorochrome [16]. This technique relies on the staining of plasmodial genomic material with DAPI followed by ultraviolet excitation of this fluorochrome and finally, microscopic detection of malaria parasites using a microscope called Cyscope® [17]. Malaria parasites are identified as luminescent small spots as compared to white blood cells (WBC) that appear bigger (Fig. 1). The Cyscope® is already using by many researchers and had been evaluated in community field for diagnosis of malaria and other diseases such as urinary schistosomiasis and gastrointestinal infections [13, 18–20]. Based on the peculiarity of this diagnostic technique, this study was conducted to investigate the performances of Cyscope® fluorescence microscope (FM) in comparison with light microscopy (LM) at the workplace. The aim was to determine if this diagnostic tool can be valuably used at the workplace as an integrative part of health strategies developed by companies to fight against malaria.

## Materials and methods

### Study sites and population

The present study is part of a larger study on malaria carried out in some companies in the town of Douala, Littoral Region, Cameroon. The conditions and characteristics of the study site have been described elsewhere [21]. Briefly, Douala represents the economic capital with 35.1% of companies of Cameroon. The climate of Douala is warm and humid with a constant temperature of about 26 °C and very heavy rainfall, especially between June and October which facilitates malaria transmission. These two reasons have guided the choice of these sites [21].

In order to preserve their notoriety, activity-guided codes were assigned to the companies involved in this study, namely, CON and BPT. These companies are based in third district of Douala. Company CON is a dealer of vehicles of several foreign brands while company BPT is focused on buildings and public works. The companies CON and BPT are endowed with an infirmary which allows the employees to manage their health problems including malaria. In addition, the companies have implemented strategies to fight against malaria as distribution of ITNs and education campaigns on malaria.

### Study design

This cross-sectional study was conducted on 21 and 22 March 2017 in two companies of the town of Douala. Prior to the field investigation, all administrative and ethical authorizations were issued by companies investigated and ethical committee of the University of Douala. After obtaining approval from the companies, members of communication cell of each company were approached in order to inform employees on the objectives and dates of the study. Employees were recruited with respect to their recent history of antimalarial drug use. Persons who have received quinine, artemisinin derivatives within the last 7 days, 4-aminoquinolines within the last 14 days, pyrimethamine and/or sulfonamides within the last 28 days, or mefloquine within the last 56 days were excluded from the study. Any employee who was working in any of these two companies and who signed informed written consent was included in the study.

The sample size of study population was calculated with regard to the following formula used for diagnostic studies as defined by Hajian-Tilaki et al. [22].

$$ N=\frac{\left[Z\frac{\alpha }{2}\ \sqrt{Po\ \left(1- Po\right)}+ Z\beta \sqrt{P1\ \left(1-P1\right)}\right]2}{{\left(P1- Po\right)}^2\ } $$

where *P*_0_ denotes the pre-determined value of sensitivity of new diagnostic test (95%); *P*_1_ is the value of sensitivity under alternative hypothesis observe in the field (98.3% ) [23]. The parameters Z_α/2_ and Z_β_ denote the percentiles of standard normal distribution and α, β are the probability of type I and type II errors respectively. In this study *α* = 0.05, Z_α/2_ is equal 1.96 and *Z*_β_ is equal 0.84, and d = accepted margin of error (5%). The estimated minimum sample size was estimated *n* = 199.7. A total of 442 employees were enrolled in this study.

### Sample and data collection

Before blood sample collection from the targeted employees, they were debriefed verbally and encouraged to sign an informed consent form before sample collection using a pre-tested structured questionnaire. Demographic data such as the name, gender, age, and auxiliary temperature of each employee were recorded before blood collection for detection of malaria parasites. The diagnosis of malaria was confirmed by both LM and FM.

### Laboratory detection

#### Fluorescence microscopy

FM was performed according to the manufacturer’s recommendation (Partec-Sysmex, Japan). Briefly, middle finger of each employee was cleaned with a cotton swab soaked in ethanol at 70°, and blood was drawn using a lancet. The first drop of blood was discarded, and the second (~ 10 μL) was deposited on a fluorochrome dried, the DAPI, on the center of the slide. The preparation is covered with a coverslip and incubated in a sunlight-free area at room temperature for 1 min. Parasites were observed at the × 40 objective of Cyscope®, and the results were interpreted as described previously [17].

#### Light microscopy

This detection technique was performed as described by Cheesbrough [24]. Two blood drops were deposited on a glass slide to make thick and thin blood smears, respectively. Thin smear was used to identify different malaria species while thick smear was used to determine parasitemia. Thin smears were fixed using absolute methanol, stained with 10% Giemsa, and then allowed to dry for 30 min. Blood drop used to make thick smear is stirred on circular motion with the corner of another glass slide and left dry for 15 min without fixative. Thick smear is then stained with 10% Giemsa, washed with buffered water , and allowed to dry for 30 min. The slides were read under immersion oil at × 100 objective lens. Parasites were counted against 200 leucocytes by assuming a mean count of 8000 leucocytes/μL for each individual [25]. Different developmental stages of *Plasmodium* were identified. Slides were read by three skilled microscopists, and the agreement between at least two of them was considered as final result of parasitological reading. Parasitemia was categorized as low (< 500 parasite/μL of blood), moderate (501–5000 parasites/μL of blood), and high (> 5000 parasites/μL of blood) according to Allen et al. [26]. Asexual parasites and gametocytes were counted against 200–500 leucocytes and converted to number of parasites per volume assuming 8000 leucocytes/μL of blood [25].

### Operational definitions

- ▪ Asymptomatic malaria defines the case of a patient diagnosed with the test but having an axillary temperature < 37.5 °C [17].
- ▪ Symptomatic malaria defines the case of a patient diagnosed as having a positive test, but having an axillary temperature ≥ 37.5 °C [17].
- ▪ True positive (TP) patients are diagnosed and positive in both tests. These are participants who have the disease and have the test is positive [13, 27].
- ▪ False positive (FP) patients are diagnosed and positive by FM but negative by ordinary microscopy. These are participants who do not have the disease but are test positive [13, 27].
- ▪ False negative (FN) participants are diagnosed and negative on FM but positive on ordinary microscopy. These are participants who have the disease but the test is negative [13, 27].
- ▪ True negative (TN) participants are the participants who are diagnosed and negative for both tests [13, 27].
- ▪ Sensitivity (Se) is the ability of the test to correctly identify those who have the disease: Se = TP/(TP + FN) [28].
- ▪ Specificity (Sp) is the ability of the test to correctly identify those who do not have the disease: Sp = TN/(TN + FP) [28].
- ▪ Positive predictive value (PPV) is the probability that a disease is present when the test is positive: PPV = TP/(TP + FP) [29].
- ▪ Negative predictive value (NPV) is the probability that a disease is absent when the test is negative: NPV = TN/(TN + FN) [30].
- ▪ Positive likelihood ratio (PLR) is the ratio between the probability of having a positive test when the person is sick and the probability of having a positive test when the person is not sick: PLR = Se/(1-Sp) [28, 31].
- ▪ Negative likelihood ratio (NLR) is the ratio between the probability of having a negative test when the person is sick and the probability of having a negative test when the person is not sick: NRL = (1-Se)/Sp [32].
- ▪ Accuracy is the probability that a patient have been correctly classified according to the result of the test: accuracy = (TP + TN)/(TP + TN + FP + FN) [33].
- ▪ Reliability of a diagnostic test depends on the accuracy and reproducibility of the test results: reliability = [(TP × TN)−(FP × FN)]/(TP + FN) × (TN + FP) [30].
- ▪ Kappa (k) values expressed the agreement beyond chance and were calculated with a 95% confidence interval. A kappa value of 0.21–0.60, 0.61–0.80, and > 0.80 was considered as moderate, good, and almost perfect agreement beyond chance, respectively [34].

### Statistical analysis

All data obtained were subjected to analysis by using the Statistical Package for Social Science (SPSS) software version 16 (SPSS Inc., Chicago, IL, USA). McNemar’s chi-square test was used to compare the prevalence of malaria infection between LM and FM. Pearson’s chi-square and non-parametric Mann-Whitney tests were used to compare unpaired proportions and mean values of parasitemia, respectively. Parasitemia was log_10_-transformed before performing statistical analysis. Statistical significance was set at *p* value < 0.05.

## Results

### Demographic characteristics of study participants

Table 1 summarizes the sociodemographic characteristics of the study population. Out of the 442 employees recruited in the study, 83.9% were male, and nearly half of the participants were aged between 19 and 35 years old (47.5%) and had completed secondary level of education (47.6%). Most employees were belonging to the worker professional category (59%) and living in the third district of Douala (63.6%). More than 87.3% of the employees diagnosed were afebrile.

**Table 1:** Baseline characteristics of study population

Parameters	Frequencies (%) or mean ± SD
**Companies**	
CON	279 (36.9)
BPT	163 (63.1)
**Age**	
[19–36[	210 (47.5)
[36–60[	200 (45.2)
≥ 60	32 (7.2)
Mean age (SD)	37.8 (9.7)
**Gender**	
Male:female ratio	3.86:1
**Fever** (axillary temperature ≥ 37.5 °C)	
Yes	56 (12.7)
No	386 (87.3)
**Level of education**	
Primary	46 (10.4)
Secondary	158 (35.7)
University	128 (29.0)
Missing data	110 (24.9)
**Professional category**	
Workers	261 (59.0)
Managers	71 (16.1)
Missing data	110 (24.9)
**Subdivision**	
Douala 1	20 (4.5)
Douala 2	17 (3.8)
Douala 3	208 (47.1)
Douala 4	15 (3.4)
Douala 5	66 (14.9)
Out of Douala	1 (0.2)
Missing data	115 (26)

*SD* Standard deviation

### Prevalence of malaria

The prevalence of malaria was 39.2% (173/441) and 17% (75/441) using FM and LM, respectively, and the difference was statistically significant between both the methods (McNemar test, *p* < 0.01). *Plasmodium falciparum* and *P. vivax* were the two species identified in this study (Table 2). All cases were mono-infection with either of the *Plasmodium* species identified; thus, the prevalence of *P. falciparum* and *P. vivax* among infection cases was 96% and 4%, respectively. In terms of developmental stages, 68% of employees diagnosed with malaria using LM carried gametocytes while trophozoites and schizonts were found in 45.3% and 1.3% of them, respectively. The geometric mean parasite density (GMPD) was 1850 parasites per μL of blood (ranges between 1600 and 40,000) while most of the employees (60.8%) had moderate parasitemia. Regarding the three *P. vivax* cases recorded, two of them had moderate parasitemia while the remaining case had low parasitemia.

**Table 2:** Malariometric variables using light microscopy

Factors	***N*** (%)
**Species**	
*P. falciparum*	72 (96)
*P. vivax*	3 (4.0)
**Stages**	
Gametocytes	51 (68.0)
Trophozoites	34 (45.3)
Schizonts	1 (1.3)
**Parasitemia (parasites/μL)**	
Low (1–500)	26 (35.1)
Moderate (501–5000)	45 (60.8)
High (> 5000)	3 (4.1) )

### Diagnostic performances of FM

Based on the results shown in Table 3, the diagnostic performances of FM were as follows: Se = 84%, Sp = 69.95%, PPV = 63.58%, NPV = 95.5%, accuracy = 89.36%, reliability = 53.95%, and kappa = 0.43, respectively. FM was not able to detect 12 cases which were positive by LM, and mean parasitemia for these cases was 170 parasites/μL.

**Table 3:** Cyscope® FM results compared to LM results with respect to clinical status

		Cyscope® FM								
		All			Febrile			Afebrile		
		Positive	Negative	Total	Positive	Negative	Total	Positive	Negative	Total
**LM**	Positive	63	12	75	4	3	7	59	9	68
	Negative	110	256	366	17	32	49	93	224	317
	**Total**	**173**	**268**	**441**^a^	**21**	**35**	**56**	**152**	**233**	**385**

*FM* Fluorescence microscopy, *LM* Light microscopy

Data are presented as frequency

^a^Parasitological result was missing for one individual

Despite the fact that the sensitivity of Cyscope® FM was lower in febrile patients as compared to their afebrile counterparts, no statistically significant difference was observed between the two groups (57.14% vs 86.16%, *p* value = 0.74) (Table 4). It should be noted that mean parasitemia was slightly higher in afebrile patients (1947 parasites/μL) compared to that of febrile patients (1029 parasites/μL).

**Table 4:** Diagnostic performances of Cyscope® FM with regard to clinical status

Parameters	All (***n*** = 441)		Febrile (***n*** = 56)		Afebrile (***n*** = 385)		***P*** value
	Value	95% CI	Value	95% CI	Value	95% CI	
Se (%)	84.00	74.08–90.60	57.14	25.04–84.18	86.76	76.71–92.87	0.74
Sp (%)	69.95	65.07–74.42	65.3	51.32–77.08	70.66	65.42–75.40	0.92
PPV (%)	63.58	56.19–70.38	19.05	7.67–40.0	38.81	31.44–46.75	0.3
NPV (%)	95.55	88.31–94.72	91.43	77.62–97.04	96.13	92.83–97.96	1
PLR	2.8	–	1.65	NA	2.95	NA	–
NLR	0.23	–	NA	NA	NA	NA	–
Accuracy (%)	89.36	83.34–90.21	10.08	51.2–75.85	79.27	68.89–77.67	0.63
Reliability (%)	53.95	–	22.45	–	57.43	–	–
Kappa	0.45	–	–	–	-	–	–

*CI* Confidence interval, *FM* Fluorescence microscopy, *Se* Sensitivity, *Sp* Specificity, *PPV* Positive predictive value, *NPV* Negative predictive value, *PLR* Positive likelihood ratio, *NLR* Negative likelihood ratio, *NA* Not applicable. Pearson’s chi-square test was used to make comparisons. Significance was set at *p* value < 0.05

Besides, sensitivity of Cyscope® microscope increased as a function of parasitemia with values ranging from 76.92% (95% CI 57.95–88.96%) in patients with parasitemia comprised between 1 and 500 parasites/μL to 91.11% (95% CI 79.27–96.49%) in patients with parasitemia comprised between 501 and 5000 parasites/μL. The difference between sensitivity values was not statistically significant (*χ*^2^ = 1.69, df = 1, *p* value = 0.19). It was not possible to compute the sensitivity for group of patients with parasitemia above 5000 parasites/μL because of small sample size for this group (*n* = 3).

## Discussion

The present study aimed at evaluating the diagnostic performances of Cyscope® FM in the detection of carriers of malaria parasites. The overall prevalence of malaria was 39.2% using FM and 17% using LM. The two malaria species involved in infection cases were *P. falciparum* and *P. vivax*. Many studies reported the circulation of *P. vivax* and its involvement in malaria burden in Cameroon [32, 35, 36].

In our study, we found 96% of malaria cases were due to *P. falciparum*, and this is line with the finding of Sandeu et al. who recorded the predominance of this species in a study conducted in Center and Southern regions of Cameroon [37, 38] and about 4.4 % greater than that which was reported by Kimbi et al. [13] in the southwest part of Cameroon [13]. *P. falciparum* is the cause of almost all malaria infections observed in Africa [2] and in particular, in Cameroon [39].

Most of infections cases were due to *P. falciparum*, and this is consistent with previous studies carried out in the Southern part [13, 18, 29] and in the Littoral region of Cameroon [17]. Briefly abovementioned, *P. vivax* was reported in the present study. Many previous molecular studies revealed the presence of this species in the country [32, 40]. This species is being considered minor in SSA due to its high level of individual deficient with red blood cell Duffy antigen, a receptor requested for the invasion of RBC by the parasite [41]. This fact is increasingly refuted as many studies reported *P. vivax* infection cases among Duffy-negative individuals in SSA especially in Cameroon [40, 42]. This suggests that either the partial role of Duffy antigen as invasion-aimed receptor or possible adaption of the parasite. Besides, the control of *P. vivax* is a veritable challenge due among others: (i) the production of dormant development stages (hypnozoites) for which all currently available antimalarial drugs, with the exception of primaquine, are inefficient, (ii) diagnosis-related problems as it may confuse with *P. ovale*, (iii) infection cases at low parasitemia that are generally revealed using molecular methods, and (iv) difficulty to cultivate this parasite [43]. In an attempt in eliminating malaria, it is of utmost importance to pay attention on non-falciparum species in fight strategies especially in SSA.

Concerning the performances of FM, we found a sensitivity value of 84% similar with the result of several other studies which also recorded values close to 95% in Southern Cameroon [13, 18, 29], in Uganda [44], Sudan [23], Ghana [34], and Ethiopia [45]. The sensitivity that we found was little less than those reported by Ndamukong-Nyanga et al. (87.6%) [18] and Kimbi et al. (91.3%) [13]. This can be explained by the difference of the study population and the area of study. Their study population was children, and the study took place in one of the rural zone in Southwest Cameroon while the present study was conducted among adults residing in an urban region. In general, malaria burden (i.e., prevalence and level of parasitemia) is higher in children and rural areas as compared to adults and urban areas, respectively [2, 46, 47]. These factors can influence the performances of malaria diagnostic tests [17]. The Cyscope® microscope gave 12 FN results which were positive with LM, and this could be due to low parasitemia. Indeed, the mean parasitemia for these 12 cases was 170 parasites/μL, and this is consistent with the finding of Sousa-Figueiredo and co-workers reported that the detection of positive cases using Cyscope® microscope was easier when parasitemia is above 400 parasites [44].

The specificity that we found (69.9%) was less than that which was found by Hassan et al. (98.3%) [23]. This difference could be related to difference in study population as can be explained by their study population which was all patients with fever reporting to the malaria Centre (Sinnar Hospital). Many researchers have shown the significant association between malaria and infection fever [48, 49]. Several false positives found can be the artifact such as dust [44] or the bacterial [50, 51] and schistosome DNA which have been seen to be confused with plasmodial DNA [34, 44]. In some cases, false positives can result in overuse of antimalarial drugs, thus unnecessarily exposing individuals to toxicity of antimalarial drugs and at larger level to increase in risk for appearance and spread of drug resistance [44].

LM, a standard technique, has the advantage that can discriminate and quantify plasmodial species [23]. However, it has some drawbacks limiting its optimal utilization in African settings. These include mainly the need for skilled microscopist, good quality reagents and microscope, and electricity which is often lacking in resource-limited and difficult-to-reach areas [17, 21, 45, 52]. In addition, the method is particularly time consuming which is not allow for prompt management of malaria cases. Finally, washing steps increase the losses of malaria parasites and thus reduce the chances to detect parasites especially in low parasitemia infections. In such situations, the implementation of LM is particularly challenging. These limitations of LM are transposable in company milieu where infirmary and health point-of-care in companies face the same difficulties to diagnose malaria. In contrast, Cyscope® may overcome the limitations of LM as the technique is rapid (~ 2 min/test), cheap, request little training, has no washing steps, and can be used in the absence of electricity as the Cyscope® is endowed with an in-built 6-h batteries. These abovementioned characteristics have been convincing its utilization by more and more authors in different contexts in Cameroon such as active case detection of malaria cases in community especially in remote areas where electricity is often lacking and passive case in health facilities [13, 17, 18]. In addition, a study showed that Cyscope® FM is cost-effective for community diagnosis of malaria as compared to immunochromatographic RDTs, quantitative buffy coat microscopy, and LM, suggesting a more important utility of Cyscope® FM in malaria endemic areas especially in SSA countries [53].

Sensitivity of Cyscope® has increased as a function of parasitemia with values ranging from 71 (1–500 parasites/μL) to 91% (501–5000 parasites/μL), and this is consistent with previous studies [29, 54]. The higher the parasitemia, the more the chances the test of interest gives a positive result.

Surprisingly, sensitivity of Cyscope® microscope was better in afebrile individuals as compared to their febrile counterparts, although no statistically significant difference was found. This can be explained by differences in level of parasitemia in these two groups as level of parasitemia was on average higher in afebrile individuals. In addition, it should be noted that fever is not a pathognomonic sign of malaria infection as infections caused by virus and bacteria can also elicit fever. Studies outlined that a large proportion of febrile illness episodes were caused by urinary tract infection bacteria mainly *Escherichia coli*, *Staphylococcus aureus*, and *Streptococcus pneumoniae* [55, 56]. In Cameroon, Achonduh-Atijegbe and colleagues reported that *Toxoplasma gondii* and *Salmonella typhii* were involved in fever cases among children [57]. Thus, some fever cases reported in the present study were of non-malarial origin, and this aspect has not been addressed. It would be interesting to address this aspect in future investigations.

The study has some limitations. First, polymerase chain reaction-based molecular method was not used as gold standard. Although this method is more sensitive than LM [23], it is not affordable for most health companies in malaria endemic areas, and their utilization is limited to research needs. Thus, the comparison of Cyscope® with LM is more logical and practical. Secondly, this study was conducted in a few companies, and the findings cannot be generalizable to all companies of Cameroon.

## Conclusion

The Cyscope® microscope has appreciable sensitive and specificity compared to the LM at the workplace. In this environment of workplace, operational characteristics of the Cyscope® will make it more adaptable at workplace than LM. The Cyscope® can therefore be recommended to all types of companies for a prompt and better diagnosis of malaria.

## Data Availability

All datasets on which the conclusions of the research rely are presented in this paper. However, data is available from the corresponding author on reasonable request.

## Abbreviations

ACT: Artemisinin-based combination therapy
ANOVA: Analysis of variance
CI: Confidence intervals
df: Degree of freedom
FN: False negative
FP: False positive
FM: Fluorescence microscopy
GMPD: Geometric mean parasite density
κ: Kappa index
LM: Light microscopy
NA: Not applicable
NLR: Negative likelihood ratio
NPV: Negative predictive values
ORs: Odds ratios
pfHRP2: *Plasmodium falciparum* histidine-rich protein 2
PLR: Positive Likelihood Ratio
PPV: Positive predictive values
RDT: Rapid diagnostic tests
SD: Standard deviation
Se: Sensitivity
Sp: Specificity
TN: True negative
TP: True positive
WBC: White blood cells
WHO: World Health Organization

## References

[1] Bonneville J, Defrance C, Miklavec T. Guide pratique de lutte contre le paludisme en entreprise. France: Sanisphere; 2007. p. 116. https://docplayer.fr/13312311-Guide-pratique-de-lutte-contre-le-paludisme-en-entreprise-j-bonneville-c-d.

[2] OMS Rapport sur le paludisme dans le monde 2019; Organisation Mondiale de la Santé: Genève, 2019; https://www.who.int/malaria/media/world-malaria-report-2019/fr/.

[3] Leighton C, Foster R. Economic impacts of malaria in Kenya and Nigeria. Research paper 6, Health financing and sustainability project 1993. http://phrplus.org/Pubs/hfsmar6.pdf.

[4] Lukwa AT, Mawoyo R, Zablon KN, Siya A, Alaba O (2019). Effect of malaria on productivity in a workplace: the case of a banana plantation in Zimbabwe. Malar J..

[5] RBM Investissement des entreprises dans la lutte contre le paludisme; Organisation Mondiale de la Santé: Genève, 2011. https://path.azureedge.net/media/documents/MCP_rbm_pi_rpt_6_fr.pdf.

[6] Nuwamanya S, Kansiime N, Aheebwe E, Akatukwasa C, Nabulo H, Turyakira E (2018). Utilization of long-lasting insecticide treated nets and parasitaemia at 6 months after a mass distribution exercise among households in Mbarara Municipality, Uganda: A cross-sectional community based study. Malar Res Treat.

[7] Zhu L, Müller GC, Marshall JM, Arheart KL, Qualls WA, Hlaing WM (2017). Is outdoor vector control needed for malaria elimination? An individual-based modelling study. Malar J.

[8] Okumu FO, Mbeyela E, Lingamba G, Moore J, Ntamatungiro AJ, Kavishe DR (2013). Comparative field evaluation of combinations of long-lasting insecticide treated nets and indoor residual spraying, relative to either method alone, for malaria prevention in an area where the main vector is *Anopheles arabiensis*. Parasites Vectors..

[9] de Jongh TE, Harnmeijer JH, Atun R, Korenromp EL, Zhao J, Puvimanasinghe J (2014). Health impact of external funding for HIV, tuberculosis and malaria: systematic review. Health Policy Plan..

[10] Guthmann JP (2009). Recherche clinique et action humanitaire: Le rôle de Médecins sans Frontières dans la lutte contre le paludisme. Med Sci..

[11] Nyasa RB, Zofou D, Kimbi HK, Kum KM, Ngu RC, Titanji VPK. The current status of malaria epidemiology in Bolifamba, atypical Cameroonian rainforest zone: an assessment of intervention strategies and seasonal variations. BMC Public Health. 2015;15. 10.1186/s12889-015-2463-1. 10.1186/s12889-015-2463-1 PMC4636784 26546458

[12] Besnard P, Foumane V, Foucher JF, Beliaud P, Costa J, Monnot N (2006). Impact de la création d’un laboratoire de diagnostic parasitologique du paludisme sur le diagnostic et le coût du paludisme dans une entreprise : une expérience angolaise. Med Trop..

[13] Kimbi HK, Ajeagah HU, Keka FC, Lum E, Nyabeyeu HN, Tonga CF (2012). Asymptomatic malaria in school children and evaluation of the performance characteristics of the Partec Cyscope® in the Mount Cameroon Region. J Bacteriol Parasitol..

[14] Shillcutt S, More C, Goodman C, Coleman P, Bell D, Whitty C (2008). Cost-effectiveness of malaria diagnostic methods in sub-Saharan Africa in an era of combination therapy. Bull World Health Organ..

[15] Wongsrichanalai C, Barcus MJ, Muth S, Sutamihardja A, Wernsdorfe WH (2007). A review of malaria diagnostic tools: microscopy and rapid diagnostic test (RDT). Am J Trop Med Hyg.

[16] Nkrumah B, Agyekum A, Acquah SEK, May J, Tannich E, Brattig N (2010). Comparison of the novel partec rapid malaria test to the conventional giemsa stain and the gold standard Real-Time PCR. J Clin MicrobioL..

[17] Lehman L, Foko L, Tonga C, Nyabeyeu H, Eboumbou E, Nono L (2018). Epidemiology of malaria using LED fluorescence microscopy among schoolchildren in Douala, Cameroon. Int J Trop Dis Health.

[18] Ndamukong-Nyanga J, Kimbi H, Sumbele I, Bertek S, Lafortune K, Larissa K (2015). Comparison of the Partec CyScope® rapid diagnostic test with light microscopy for malaria diagnosis in Rural Tole, Southwest Cameroon. British J Med Med Res..

[19] Wepnje GB, Anchang-Kimbi JK, Ndassi VD, Lehman LG, Kimbi HK (2019). Schistosoma haematobium infection status and its associated risk factors among pregnant women in Munyenge, South West Region, Cameroon following scale-up of communal piped water sources from 2014 to 2017: a cross-sectional study. BMC Public Health..

[20] Lehman LG, Kouodjip Nono L, Bilong Bilong CF (2012). Diagnostic des parasitoses intestinales à l’aide de la microscopie à fluorescence. Med Afr Noire..

[21] Mbohou CN, Kojom Foko LP, Nyabeyeu HN, Tonga C, Nono LK, Kangam L (2019). Malaria screening at the workplace in Cameroon. PLoS One..

[22] Hajian-Tilaki K (2014). Sample size estimation in diagnostic test studies of biomedical informatics. J Biomed Inform..

[23] Hassan SEDH, Okoued SI, Mudathir MA, Malik EM. Testing the sensitivity and specificity of the fluorescence microscope (Cyscope®) for malaria diagnosis. Malar J. 2010;9. 10.1186/1475-2875-9-88. 10.1186/1475-2875-9-88 PMC2860357 20356369

[24] Cheesbrough M. District laboratory practice in tropical countries: Cambridge University Press; 2006. p. 117. ISBN 13 978-0-511-34842-6.

[25] Trape JF. Rapid evaluation of malaria parasite density and standardization of thick smear examination for epidemiological investigations. The United Kingdom: Trans R Soc Trop Med Hyg. 1985;79:181–4. 10.1016/0035-9203(85)90329-3. 10.1016/0035-9203(85)90329-3 3890280

[26] Allen S, Ennett S, Riley E, Rowe P, Jakobsen P, Donnell A (1992). Morbidity from malaria and immune responses to defined *Plasmodium falciparum* antigens in children with sickle cell trait in the Gambia. Trans R Soc Trop Med Hyg..

[27] Yavo W, Ackra KN, Menan EIH, Barro-Kiki PC, Kassi RR, Adjetey TAK (2002). Étude comparative de quatre techniques de diagnostic biologique du paludisme utilisées en Côte d’Ivoire. Bull Soc Pathol Exot..

[28] Perneger TV, Szeless T, Rougemont A (2006). Utility of the detection of *Plasmodium* parasites for the diagnosis of malaria in endemic areas. BMC Infect Dis..

[29] Wanji S, Kimbi HK, Eyong JE, Tendongfor N, Ndamukong JL. Performance and usefulness of the Hexagon rapid diagnostic test in children with asymptomatic malaria living in the Mount Cameroon region. Malar J. 2008;7. 10.1186/1475-2875-7-89. 10.1186/1475-2875-7-89 PMC2427036 18498626

[30] Jaeschke R, Guyatt G, Sackett D (1994). Users’ Guides to the Medical Literature: III. How to use an article about a diagnostic test: B. What are the results and will they help me in caring for my patients?. JAMA.

[31] Wanja EW, Kuya N, Moranga C, Hickman M, Johnson JD, Moseti C (2016). Field evaluation of diagnostic performance of malaria rapid diagnostic tests in western Kenya. Malar J..

[32] Fru-Cho J, Bumah VV, Safeukui I, Nkuo-Akenji T, Titanji VP, Haldar K (2014). Molecular typing reveals substantial *Plasmodium vivax* infection in asymptomatic adults in a rural area of Cameroon. Malar J..

[33] Kalungu M, Mbabazi P, Hopkins H, Byakika-Kibwika P, Osilo E, Kamya MR (2015). Accuracy of two malaria rapid diagnostic tests (RDTS) for initial diagnosis and treatment monitoring in a high transmission setting in Uganda. Am J Trop Med Hyg..

[34] Nkrumah B, Acquah SE, Ibrahim L, May J, Brattig N, Tannich E, et al. Comparative evaluation of two rapid field tests for malaria diagnosis: Partec Rapid Malaria Test® and Binax Now® Malaria Rapid Diagnostic Test. BMC Infect Dis. 2011;11. 10.1186/1471-2334-11-143. 10.1186/1471-2334-11-143 PMC3118144 21605401

[35] Russo G, Faggioni G, Paganotti GM, Djeunang Dongho GB, Pomponi A, De Santis R (2017). Molecular evidence of *Plasmodium vivax* infection in Duffy negative symptomatic individuals from Dschang, West Cameroon. Malar J.

[36] Mbenda HGN, Awasthi G, Singh PK, Gouado I, Das A (2014). Does malaria epidemiology project Cameroon as ‘Africa in miniature’?. J Biosci..

[37] Sandeu MM, Bayibéki AN, Tchioffo MT, Abate L, Gimonneau G, Awono-Ambéné PH (2017). Do the venous blood samples replicate malaria parasite densities found in capillary blood? A field study performed in naturally-infected asymptomatic children in Cameroon. Malar J.

[38] Sundararaman SA, Liu W, Keele BF, Learn GH, Bittinger K, Mouacha F (2013). Plasmodium falciparum-like parasites infecting wild apes in southern Cameroon do not represent a recurrent source of human malaria. PNAS..

[39] Antonio-Nkondjio C, Ndo C, Njiokou F, Bigoga JD, Awono-Ambene P, Etang J (2019). Review of malaria situation in Cameroon: technical viewpoint on challenges and prospects for disease elimination. Parasites Vectors..

[40] Ngassa Mbenda HG, Das A (2014). Molecular evidence of *Plasmodium vivax* mono and mixed malaria parasite infections in duffy-negative native Cameroonians. PLoS One..

[41] Twohig KA, Pfeffer DA, Baird JK, Price RN, Zimmerman PA, Hay SI (2019). Growing evidence of *Plasmodium vivax* across malaria-endemic Africa. PLoS Negl Trop Dis..

[42] Ngassa Mbenda HG, Gouado I, Das A (2016). An additional observation of Plasmodium vivax malaria infection in Duffy-negative individuals from Cameroon. J Infect Dev Ctries..

[43] Gunalan K, Niangaly A, Thera MA, Doumbo OK, Miller LH (2018). *Plasmodium vivax* infections of duffy-negative erythrocytes: historically undetected or a recent adaptation?. Trends parasitol..

[44] Sousa-Figueiredo JC, Oguttu D, Adriko M, Besigye F, Nankasi A, Arinaitwe M (2010). Investigating portable fluorescent microscopy (CyScope®) as an alternative rapid diagnostic test for malaria in children and women of child-bearing age. Malar J..

[45] Birhanie M (2016). Comparison of Partec rapid malaria test with conventional light microscopy for diagnosis of malaria in Northwest Ethiopia. J Parasitol Res..

[46] Maghendji-Nzondo S, Kouna LC, Mourembou G, Boundenga L, Imboumy-Limoukou RK (2016). Matsiegui, et al. Malaria in urban, semi-urban and rural areas of southern of Gabon: comparison of the *Pfmdr* 1 and *Pfcrt* genotypes from symptomatic children. Malar J..

[47] Kabaria CW, Gilbert M, Noor AM, Snow RW, Linard C (2017). The impact of urbanization and population density on childhood *Plasmodium falciparum* parasite prevalence rates in Africa. Malar J..

[48] Okiro EA, Snow RWT (2010). Relationship between reported fever and *Plasmodium falciparum* infection in African children. Malar J.

[49] Iroezindu MO, Agaba EI, Okeke EN, Daniyam CA, Isa SE, Akindigh MT (2012). Relationship between fever and malaria parasitaemia in adults: does HIV infection make any difference?. J Med Trop.

[50] Okalla E, Adiogob A, Beyiha G (2014). Profil bactériologique et sensibilité aux antibiotiques des isolats d’hémoculture (2006 – 2011) à Douala, Cameroun.

[51] Essomba NE, Ngaba GP, Koum DCK, Momo L, Coppieters Y (2015). Prévalence du Cytomégalovirus chez les Donneurs de Sang d’un Hôpital de District Urbain à Douala- Cameroun. Health Sci Dis..

[52] Kamgain L, Assam-Assam JP, Kojom Foko LP, Fouamno H (2017). Prevalence of malaria infection and reliability of ACCUCARE One Step Malaria Test® for diagnosing malaria in people living with human immunodeficiency virus infection in Cameroon. Int J Trop Dis Health..

[53] Ogunniyi A, Dairo MD, Dada-Adegbola H, Ajayi IO, Olayinka A, Oyibo WA, et al. Cost-effectiveness and validity assessment of Cyscope microscope, quantitative buffy coat microscope, and rapid diagnostic kit for malaria diagnosis among clinic attendees in Ibadan, Nigeria. Malar Res Treat. 2016:1–7. 10.1155/2016/5242498. 10.1155/2016/5242498 PMC4963599 27493827

[54] Tahar R, Sayang C, Ngane V, Soulac FG, Moyou-Somo R, Delmont J (2013). Field evaluation of rapid diagnostic tests for malaria in Yaounde, Cameroon. Acta Trop.

[55] Dalrymple U, Cameron E, Arambepola R, Battle KE, Chestnutt EG, Keddie SH (2019). The contribution of non-malarial febrile illness co-infections to *Plasmodium falciparum* case counts in health facilities in sub-Saharan Africa. Malar J..

[56] Pondei K, Kunle-Olowu OE, Peterside O (2013). The aetiology of non-malarial febrile illness in children in the malaria-endemic Niger Delta Region of Nigeria. Asian Pac J Trop. Dis..

[57] Achonduh-Atijegbe OA, Mfuh KO, Mbange AHE, Chedjou JP, Taylor DW, Nerurkar VR (2016). Prevalence of malaria, typhoid, toxoplasmosis and rubella among febrile children in Cameroon. BMC Infect Dis..

